# Supplementary material for: BIChromET: A Chromogenic Culture Medium for Detection of Piperacillin/Tazobactam and Cefepime Resistance in Pseudomonas aeruginosa
Source: Antibiotics (Basel). 2023 Oct 28;12(11):1573. doi: 10.3390/antibiotics12111573 (PMC10668787; doi:10.3390/antibiotics12111573)
Supplement: Supplementary file 1 [file antibiotics-12-01573-s001.zip › antibiotics-2675634-supplementary.pdf]

Table S1. Raw data of the BIChromET evaluation step for cefepime.

Isolates	Source	1,00E+04	1,00E+05	1,00E+06	1,00E+07	1,00E+08	CMI FEP
C-		N	N	N	N	N	2
C+		Y	Y	Y	Y	Y	256
PAS1	Blood	N	N	N	N	N	4
PAS2	Blood	N	N	N	N	N	4
PAS3	Blood	N	N	N	N	N	2
PAS4	Blood	N	N	N	N	N	2
PAS5	Blood	N	N	N	N	N	2
PAS6	Urine	N	N	N	N	N	2
PAR82	Sputum	N	N	N	N	N	4
PAR83	Sputum	Y	Y	Y	Y	Y	32
PAR84	Cutaneous abscess	Y	Y	Y	Y	Y	64
PAR1	BAL	Y	Y	Y	Y	Y	32
PAR3	TBA	Y	Y	Y	Y		>256
PAR4	TBA	Y	Y	Y	Y	Y	16
PAR5	TBA	Y	Y	Y	Y	Y	16
PAR6	Blood	N	N	N	N	N	4
PAR7	TBA	Y	Y	Y	Y	Y	16
PAR9	TBA	Y	Y	Y	Y	Y	128
PAR19	Corneal scraping	N	N	N	N	N	4
PAR21	TBA	N	N	N	N	N	8

PAR20	TBA	N	N	N	N	N	8
PAR26	Wound	N	N	N	N	N	8
PAR32	TBA	Y	Y	Y	Y	Y	64
PAR33	TBA	N	N	N	N	N	8
PAR36	Urine	N	N	N	N	N	2
PAR35	Urine	Y	Y	Y	Y	Y	128
PAR40	Wound	N	N	N	N	N	8
PAR42	Urine	N	N	N	N	N	8
PAR44	Wound	Y	Y	Y	Y	Y	32
PAR46	Blood	N	N	N	N	N	4
PAR47	TBA	Y	Y	Y	Y	Y	16
PAR49	TBA	Y	Y	Y	Y	Y	16
PAR50	Wound	N	Y	Y	Y	Y	16
PAR51	Wound	Y	Y	Y	Y	Y	64
PAR52	Wound	N	N	N	N	Y	8
PAR53	CSF	N	N	N	N	N	4
PAR54	Blood	N	Y	Y	Y	Y	16
PAR55	TBA	N	N	N	N	N	2
PAR56	Blood	N	N	Y	Y	Y	16
PAR57	BAL	Y	Y	Y	Y	Y	32
PAR58	Wound	Y	Y	Y	Y	Y	32
PAR59	TBA	N	N	N	N	Y	8

PAR60	TBA	Y	Y	Y	Y	Y	16
PAR61	TBA	N	N	N	N	N	4
PAR62	Blood	N	N	Y	Y	Y	16
PAR65	Urine	N	N	N	N	N	2
PAR66	Bile	Y	Y	Y	Y	Y	32
PAR72	Blood	N	N	N	N	N	8
PAR73	Wound	N	N	N	N	N	8
PAR74	Blood	Y	Y	Y	Y	Y	32
PAR85	Wound	Y	Y	Y	Y	Y	64
PAR86	Wound	N	N	N	N	N	0.5
PAR87	TBA	N	N	N	N	N	8
PAR88	Sputum	N	N	N	N	N	8
PAR89	TBA	N	N	N	N	N	2
PAR90	TBA	N	N	N	N	N	8
PAR91	TBA	N	N	Y	Y	Y	16
PAR92	Blood	N	N	N	N	N	8
PAR93	TBA	N	N	N	N	Y	8
PAR94	TBA	Y	Y	Y	Y	Y	64
PAR95	TBA	N	Y	Y	Y	Y	16
PAR96	Blood	N	N	N	N	N	4
PAR97	BAL	Y	Y	Y	Y	Y	64
PAR98	TBA	N	N	N	N	N	4

PAR99	Cutaneous abscess	Y	Y	Y	Y	Y	16
PAR100	Blood	Y	Y	Y	Y	Y	32
PAR101	TBA	N	N	N	N	N	8
PAR102	TBA	N	N	N	N	N	4
PAR103	TBA	N	N	N	N	N	8
PAR104	Wound	Y	Y	Y	Y	Y	128
PAR105	BAL	N	N	N	N	Y	8
PAR106	Sputum	N	Y	Y	Y	Y	16
PAR107	Wound	N	Y	Y	Y	Y	16
PAR108	TBA	N	N	N	N	N	2
PAR109	BAL	N	N	N	N	N	8
PAR110	TBA	Y	Y	Y	Y	Y	32
PAR111	TBA	N	N	N	N	N	4
PAR112	TBA	N	N	N	N	N	4
PAR113	TBA	Y	Y	Y	Y	Y	32
PAR114	Wound	N	N	N	N	N	8
PAR115	TBA	N	N	N	Y	Y	8
PAR116	Wound	N	N	N	N	N	4
PAR117	TBA	N	Y	Y	Y	Y	16
PAR118	Urine	N	N	N	N	N	4
PAS7	Urine	Y	Y	Y	Y	Y	16
PAS8	Urine	N	N	N	N	N	2

PAS9	Urine	N	N	N	N	N	2
PAS10	Urine	N	N	N	N	N	8
PAS11	Blood	N	N	N	N	N	2
PAS12	Urine	N	N	N	N	N	2
PAS13	Urine	N	N	N	N	N	1
PAS14	Urine	N	N	N	N	N	2
PAS15	Sputum	N	N	N	N	N	2
PAS16	TBA	N	N	N	N	N	2
PAS17	Wound	N	Y	Y	Y	Y	32
PAS18	TBA	N	N	N	N	Y	8
PAR12	BAL	Y	Y	Y	Y	Y	64
PAR16	Wound	N	Y	Y	Y	Y	16
PAR17	BAL	N	N	N	N	N	2
PAR75	CSF	N	N	N	N	N	1

BAL, bronchoalveolar lavage; TBA, tracheobronchial aspirate; CSF, cerebrospinal fluid; Y, yes; N, No; C-, negative control; C+, positive control; MIC, minimum inhibitory concentration; FEP: cefepime

Table S2. Raw data of the BIChromET evaluation step for piperacillin/tazobactam.

STRAINS	SOURCE	1,00E+04	1,00E+05	1,00E+06	1,00E+07	1,00E+08	CMI TZP
C-		N	N	N	N	N	1
C+		Y	Y	Y	Y	Y	256
PAS1	Blood	N	N	N	N	N	4
PAS2	Blood	N	N	N	N	N	8
PAS3	Blood	N	N	N	N	N	4
PAR82	Sputum	N	N	N	N	N	1
PAR83	Sputum	N	N	N	N	N	16
PAR84	Cutaneous abscess	Y	Y	Y	Y	Y	64
PAR85	Wound	Y	Y	Y	Y	Y	256
PAS4	Blood	N	N	N	N	N	2
PAS5	Blood	N	N	N	N	N	4
PAS6	Urine	N	N	N	N	N	4
PAS7	Urine	Y	Y	Y	Y	Y	32
PAS8	Urine	N	N	N	N	N	2
PAS9	Urine	N	N	N	N	N	4
PAS10	Urine	N	N	N	N	N	8
PAR1	BAL	Y	Y	Y	Y	Y	>256
PAR3	TBA	Y	Y	Y	Y	Y	>256
PAR 4	TBA	N	N	N	N	Y	16
PAR5	TBA	N	N	N	N	N	16
PAR6	Blood	N	N	N	N	N	8

PAR7	TBA	N	N	Y	Y	Y	32
PAR9	TBA	N	N	N	N	N	2
PAS11	Blood	N	N	N	N	N	4
PAS12	Urine	N	N	N	N	N	8
PAS13	Urine	N	N	N	N	N	2
PAS14	Urine	N	N	N	N	N	4
PAS15	Sputum	N	N	N	N	N	2
PAS16	TBA	N	N	N	N	N	8
PAS17	Wound	Y	Y	Y	Y	Y	64
PAS18	TBA	N	N	N	N	N	16
PAR12	BAL	N	N	N	N	N	16
PAR16	Wound	Y	Y	Y	Y	Y	>256
PAR17	BAL	N	N	N	N	N	4
PAR19	Corneal scraping	N	N	N	N	N	8
PAR21	TBA	N	N	N	N	N	16
PAR20	TBA	N	N	N	N	N	16
PAR26	Wound	N	N	N	N	N	8
PAR32	TBA	N	N	N	N	N	8
PAR33	TBA	N	N	N	N	N	8
PAR36	Urine	N	N	N	N	N	8
PAR35	Urine	N	N	N	N	N	16
PAR40	Wound	Y	Y	Y	Y	Y	256
PAR42	Urine	N	N	N	N	N	8

PAR44	Wound	Y	Y	Y	Y	Y	256
PAR46	Blood	N	N	N	N	N	16
PAR47	TBA	N	N	N	N	N	16
PAR49	TBA	Y	Y	Y	Y	Y	>256
PAR50	Wound	Y	Y	Y	Y	Y	>256
PAR51	Wound	Y	Y	Y	Y	Y	>256
PAR52	Wound	N	N	N	N	Y	16
PAR53	CSF	N	N	N	N	N	8
PAR54	Blood	Y	Y	Y	Y	Y	>256
PAR55	TBA	N	N	N	N	N	4
PAR56	Blood	N	N	N	N	N	16
PAR57	BAL	Y	Y	Y	Y	Y	>256
PAR58	Wound	Y	Y	Y	Y	Y	>256
PAR59	TBA	N	N	N	N	N	8
PAR60	TBA	Y	Y	Y	Y	Y	>256
PAR61	TBA	N	N	N	N	N	16
PAR62	Blood	Y	Y	Y	Y	Y	>256
PAR65	Urine	N	N	N	N	N	4
PAR66	Bile	Y	Y	Y	Y	Y	>256
PAR72	Blood	N	N	N	N	N	8
PAR73	Wound	N	N	N	N	N	8
PAR74	Blood	Y	Y	Y	Y	Y	>256
PAR85	Wound	Y	Y	Y	Y	Y	>256

PAR86	Wound	N	N	N	N	N	2
PAR87	TBA	N	N	N	N	N	8
PAR88	Sputum	N	N	N	N	N	8
PAR89	TBA	N	N	N	N	N	4
PAR90	TBA	N	N	N	N	N	16
PAR91	TBA	N	Y	Y	Y	Y	32
PAR92	Blood	N	Y	Y	Y	Y	32
PAR93	TBA	N	N	N	N	N	2
PAR94	TBA	Y	Y	Y	Y	Y	256
PAR95	TBA	N	N	N	N	N	16
PAR96	Blood	N	N	N	N	N	4
PAR97	BAL	Y	Y	Y	Y	Y	256
PAR98	TBA	N	N	N	N	N	4
PAR99	Cutaneous abscess	Y	Y	Y	Y	Y	256
PAR100	Blood	Y	Y	Y	Y	Y	128
PAR101	TBA	N	N	N	N	N	8
PAR102	TBA	N	N	N	N	N	8
PAR103	TBA		Y	Y	Y	Y	32
PAR104	Wound	Y	Y	Y	Y	Y	64
PAR105	BAL	N	N	N	N	N	1
PAR106	Sputum	N	N	N	N	N	4
PAR107	Wound	N	N	N	N	N	4
PAR108	TBA	N	N	N	N	N	2

PAR109	BAL	N	N	N	N	N	8
PAR110	TBA	N	N	N	N	Y	16
PAR111	TBA	N	N	N	N	N	4
PAR112	TBA	N	N	N	N	N	4
PAR113	TBA	N	N	N	N	N	16
PAR114	Wound	N	N	N	N	N	8
PAR115	TBA	N	N	N	N	Y	16
PAR116	Wound	N	N	N	N	N	4
PAR117	TBA	N	N	N	N	N	8
PAR118	Urine	N	N	N	N	N	2

BAL, bronchoalveolar lavage; TBA, tracheobronchial aspirate; CSF, cerebrospinal fluid; Y, yes; N, No; C-, negative control; C+, positive control; MIC, minimum inhibitory concentration; TZP: piperacillin/tazobactam

Table S3. Raw data of the BIChromET clinical evaluation with 173 clinical specimens (TBA and BAL)

NUMBER	ST[A]	TREATMENT[B]	BACTERIAL CONCENTRATION (CFU/ml)	BIChromET		REPORT (CONVENTIONAL TECHNIQUES)	CONFIRMATORY ANALYSIS	MIC (BMD[C])	
				FEP	TZP	IDENTIFICATION OF THE SPECIES BY MALDI-TOF	IDENTIFICATION OF THE COLONIES FROM BIChromET	FEP	TZP
BC-1	TBA		-	-	-	-			
BC-2	BAL		> 10^6	-	-	*Escherichia coli*			
BC-3	BAL		-	-	-	-			
BC-4	TBA		-	-	-	-			
BC-5	TBA		> 10^6	-	-	*Staphylococcus aureus*			
BC-6	TBA		> 10^6	-	-	*Staphylococcus aureus*			
BC-7	BAL		> 10^6	-	-	*Escherichia coli*			
BC-8	BAL		> 10^6	+	+	*Stenotrophomonas maltophilia*			
BC-9	BAL		> 10^6	+	+	*Acinetobacter baumannii*			
BC-10	TBA		-	-	-	-			
BC-11	BAL		-	-	-	-			
BC-12	TBA		-	+	+	-	*Myroides odoratus*		
BC-13	TBA		-	-	-	-			
BC-14	TBA		-	-	-	-			
BC-15	TBA		-	-	-	-			
BC-16	TBA		-	-	-	-			
BC-17	TBA		> 10^6	-	-	*Enterobacter cloacae*			
**BC-18**	**TBA**	**-**	**> 10^6**	+	+	***Pseudomonas aeruginosa***		**16**	**64**
BC-19	BAL		-	-	-	-			

BC-20	TBA		-	-	-	-			
BC-21	TBA		-	-	-	-			
BC-22	TBA		-	-	-	-			
BC-23	TBA		-	-	-	-			
BC-24	TBA		-	-	-	-			
BC-25	TBA		-	-	-	-			
BC-26	TBA		> 10^6	+	+	*Stenotrophomonas maltophilia*			
**BC-27**	**TBA**	**Amoxicillin-Clavulanic acid**	**> 10^6**	**+**	**+**	***Pseudomonas aeruginosa***	***Stenotrophomonas maltophilia***	**2**	**2**
BC-28	TBA		> 10^6	-	+	*Escherichia coli*			
BC-29	TBA		> 10^6	+	+	*Klebsiella aerogenes*	*Klebsiella aerogenes*		
BC-30	BAL		> 10^6	-	-	*Escherichia coli*			
**BC-31**	**TBA**	**Vancomycin**	**10^5 - 10^6**	**-**	**-**	***Pseudomonas aeruginosa***		**4**	**2**
BC-32	TBA		-	-	-	-			
BC-33	TBA		-	-	-	-			
BC-34	TBA		-	-	-	-			
BC-35	TBA		> 10^6	-	-	*Haemophilus influenzae*			
BC-36	TBA		-	-	-	-			
BC-37	TBA		-	-	-	-			
BC-38	TBA		10^5 - 10^6	-	-	*Klebsiella pneumoniae*			
BC-39	TBA		> 10^6	-	-	*Klebsiella pneumoniae/Staphylococcus aureus*			
**BC-40**	**TBA**	**Amphotericin B**	**> 10^6**	**+**	**+**	***Pseudomonas aeruginosa***		**16**	**128**
BC-41	TBA		> 10^6	+	-	*Klebsiella pneumoniae*			
BC-42	TBA		> 10^6	-	-	*Haemophilus influenzae*			

BC-43	TBA		> 10^6	+	+	*Stenotrophomonas maltophilia/Enterobacter cloacae*	*Stenotrophomonas maltophilia*		
BC-44	TBA		-	-	-	-			
BC-45	TBA		> 10^6	-	-	-			
BC-46	TBA		-	-	-	-			
BC-47	TBA		-	-	-	-			
BC-48	TBA		-	-	-	-			
BC-49	TBA		-	-	-	-			
BC-50	TBA		-	-	-	-			
BC-51	TBA		-	-	-	-			
BC-52	TBA		> 10^6	+	-	*Klebsiella aerogenes*			
BC-53	TBA		> 10^6	+	+	*Acinetobacter baumannii*			
BC-54	TBA		> 10^6	-	-	*Yeast*			
BC-55	BAL		-	-	-	-			
BC-56	TBA		-	-	-	-			
BC-57	TBA		> 10^6	-	-	*Raoultella ornithinolytica*			
BC-58	TBA		-	-	-	-			
BC-59	TBA		-	-	-	-			
**BC-60**	**TBA**	**-**	**> 10^6**	**-**	**-**	***Pseudomonas aeruginosa***		**1**	**2**
BC-61	TBA		> 10^6	+	+	*Escherichia coli*	*Klebsiella pneumoniae*		
BC-62	TBA		> 10^6	-	-	*Staphylococcus aureus*			
BC-63	TBA		> 10^6	-	-	*Haemophilus influenzae*			
**BC-64**	**TBA**	**Ceftazidime**	**10^5 - 10^6**	**-**	**-**	***Pseudomonas aeruginosa***		**2**	**4**
BC-65	TBA		-	-	-	-			

BC-66	TBA		-	-	-	-			
BC-67	TBA		-	-	-	-			
BC-68	TBA		-	-	-	-			
BC-69	TBA		-	-	-	-			
**BC-70**	**TBA**	**Piperacillin-Tazobactam**	**> 10^6**	**+**	**+**	***Pseudomonas aeruginosa***		**32**	**256**
BC-71	TBA		> 10^6	+	+	*Acinetobacter baumannii*			
BC-72	TBA		> 10^6	-	+	*Serratia marcescens*	*Klebsiella oxytoca*		
**BC-73**	**TBA**	**Piperacillin-Tazobactam**	**> 10^6**	**+**	**+**	***Pseudomonas aeruginosa***		**16**	**128**
BC-74	TBA		> 10^6	-	-	*Staphylococcus aureus*			
BC-75	TBA		-	-	-	-			
BC-76	TBA		-	-	-	*Yeast*			
BC-77	TBA		-	-	-	-			
BC-78	TBA		10^5 - 10^6	-	-	*Staphylococcus aureus*			
BC-79	TBA		-	-	-	-			
BC-80	TBA		10^5 - 10^6	-	-	*Enterobacter cloacae*			
BC-81	TBA		-	-	-	-			
BC-82	BAL		10^5 - 10^6	-	-	*Staphylococcus aureus*			
BC-83	TBA		10^5 - 10^6	+	+	*Stenotrophomonas maltophilia*			
**BC-84**	**TBA**	**Meropenem**	**> 10^6**	**+**	**+**	***Pseudomonas aeruginosa***		**32**	**64**
BC-85	TBA		-	-	-	-			
BC-86	TBA		-	-	-	-			
BC-87	TBA		-	-	-	-			

BC-88	TBA		> 10^6	-	-	*Escherichia coli*			
**BC-89**	**TBA**	**Ceftriaxone**	**> 10^6**	**+**	**+**	***Pseudomonas aeruginosa/Klebsiella pneumoniae***	***Klebsiella pneumoniae***	**2**	**4**
BC-90	TBA		-	-	-	-			
BC-91	TBA		-	-	-	-			
BC-92	BAL		-	-	-	-			
BC-93	TBA		10^5 - 10^6	-	-	*Escherichia coli*			
BC-94	TBA		> 10^6	-	+	*Klebsiella pneumoniae*			
BC-95	TBA		> 10^6	+	+	*Enterobacter cloacae/Acinetobacter baumannii*	*Enterobacter cloacae/Acinetobacter baumannii*		
BC-96	TBA		-	-	-	-			
BC-97	BAL		-	-	-	-			
**BC-98**	**TBA**	**-**	**10^5 - 10^6**	**-**	**-**	***Staphylococcus aureus/Pseudomonas aeruginosa***		**2**	**4**
**BC-99**	**TBA**	**Meropenem**	**> 10^6**	**+**	**+**	***Pseudomonas aeruginosa***		**32**	**128**
**BC-100**	**TBA**	**Vancomycin + Caspofungin**	**> 10^6**	**+**	**+**	***Pseudomonas aeruginosa***		**32**	**256**
BC-101	TBA		-	-	-	-			
BC-102	BAL		-	-	-	-			
BC-103	TBA		-	-	-	-			
BC-104	TBA		> 10^6	-	-	*Haemophilus influenzae/Staphylococcus aureus*			
BC-105	TBA		-	-	-	-			
BC-106	BAL		> 10^6	-	-	*Enterobacter cloacae*			
BC-107	TBA		-	-	-	-			
BC-108	BAL		-	-	-	-			

**BC-109**	**BAL**	**Meropenem + Vancomycin**	**> 10^6**	**-**	**-**	***Pseudomonas aeruginosa***		**2**	**4**
BC-110	TBA		> 10^6	-	-	*Enterobacter cloacae*			
BC-111	TBA		10^5 - 10^6	+	+	*Klebsiella pneumoniae*			
BC-112	TBA		10^5 - 10^6	-	-	*Streptococcus dysgalactiae*			
BC-113	TBA		-	-	-	-			
BC-114	TBA		> 10^6	+	+	*Stenotrophomonas maltophilia*			
BC-115	TBA		> 10^6	+	+	*Stenotrophomonas maltophilia*			
BC-116	TBA		> 10^6	-	-	*Moraxella catarrhalis*			
BC-117	TBA		> 10^6	+	+	*Acinetobacter baumannii*			
BC-118	BAL		-	-	+	-	*Klebsiella oxytoca*		
BC-119	TBA		-	-	-	-			
BC-120	TBA		-	-	-	-			
BC-121	TBA		-	-	-	-			
**BC-122**	**TBA**	**Cefepime + Vancomycin**	**10^5 - 10^6**	**-**	**-**	***Pseudomonas aeruginosa***		**4**	**16**
BC-123	TBA		-	-	-	-			
BC-124	TBA		10^5 - 10^6	-	-	*Escherichia coli*			
BC-125	TBA		-	-	-	-			
BC-126	TBA		10^5 - 10^6	-	-	*Escherichia coli/Staphylococcus aureus*			
**BC-127**	**TBA**	**Piperacillin-Tazobactam + Vancomycin**	**> 10^6**	**-**	**-**	***Pseudomonas aeruginosa***		**8**	**16**
BC-128	BAL		10^5 - 10^6	-	+	*Yeast*	*Escherichia coli*		
**BC-129**	**TBA**	**-**	**10^5 - 10^6**	**+**	**+**	***Pseudomonas aeruginosa***		**8**	**32**

BC-130	BAL		-	-	-	-			
**BC-131**	**TBA**	**Cloxacillin + Ceftazidime**	**> 10^6**	**+**	**+**	***Pseudomonas aeruginosa***		**16**	**64**
BC-132	TBA		-	-	-	-			
**BC-133**	**TBA**	**Cloxacillin + Ceftazidime**	**> 10^6**	**+**	**+**	***Pseudomonas aeruginosa***		**8**	**32**
BC-134	TBA		-	-	-	-			
**BC-135**	**TBA**	**Piperacillin-Tazobactam**	**> 10^6**	**+**	**+**	***Stenotrophomonas maltophilia/Pseudomonas aeruginosa***	***Stenotrophomonas maltophilia***	**8**	**2**
BC-136	TBA		> 10^6	-	-	*Corynebacterium argentoratense*			
BC-137	TBA		-	-	-	-			
BC-138	TBA		-	-	-	-			
BC-139	BAL		-	-	-	-			
BC-140	TBA		-	-	-	-			
BC-141	TBA		-	-	-	-			
**BC-142**	**TBA**	**Ceftzidime + Vamcomycin**	**> 10^6**	**+**	**+**	***Pseudomonas aeruginosa***		**32**	**256**
BC-143	TBA		-	-	-	-			
**BC-144**	**TBA**	**Meropenem**	**> 10^6**	**+**	**+**	***Acinetobacter baumannii/Pseudomonas aeruginosa***	***Acinetobacter baumannii (TZP+FEP)/Pseudomonas aeruginosa (TZP)***	**8**	**64**
BC-145	TBA		-	-	-	-			
BC-146	TBA		-	-	-	-			
BC-147	TBA		-	-	-	-			
BC-148	TBA		-	-	-	-			
BC-149	TBA		-	-	-	-			

BC-150	TBA		-	-	-	-		
**BC-151**	**TBA**	**-**	**> 10^6**	+	+	***Pseudomonas aeruginosa***	**32**	**256**
**BC-152**	**TBA**	**Cloxacillin + Ceftazidime**	**> 10^6**	+	+	***Pseudomonas aeruginosa***	**64**	**>256**
BC-153	TBA		-	-	-	-		
BC-154	TBA		> 10^6	-	+	*Escherichia coli*		
BC-155	TBA		10^5 - 10^6	-	-	*Enterobacter cloacae/Streptococcus constellatus*		
BC-156	TBA		10^5 - 10^6	-	-	*Enterobacter cloacae*		
BC-157	TBA		-	-	-	-		
**BC-158**	**TBA**	**-**	**> 10^6**	+	+	***Pseudomonas aeruginosa***	**16**	**256**
**BC-159**	**TBA**	**Cloxacillin + Ceftazidime**	**10^5 - 10^6**	+	+	***Pseudomonas aeruginosa***	**32**	**>256**
BC-160	TBA		> 10^6	-	-	*Haemophilus influenzae*		
**BC-161**	**TBA**	**Piperacillin-Tazobactam + Vancomycin**	**10^5 - 10^6**	+	+	***Pseudomonas aeruginosa***	**16**	**128**
BC-162	TBA		> 10^6	-	-	*Haemophilus influenzae*		
BC-163	TBA		-	-	-	-		
BC-164	BAL		-	-	-	-		
BC-165	TBA		-	-	-	-		
BC-166	TBA		-	-	-	-		
BC-167	TBA		-	-	-	-		
BC-168	TBA		-	-	-	-		
BC-169	TBA		-	-	-	-		
BC-170	TBA		10^5 - 10^6	-	-	*Streptococcus oneumoniae*		

BC-171	TBA	> 10^6	-	-	*Enterococcus faecalis*
BC-172	TBA	-	-	-	-
BC-173	TBA	-	-	-	-

TBA, tracheobronchial aspirate; BAL, bronchoalveolar lavage; FEP, cefepime; TZP, piperacillin/tazobactam; S, susceptible; R, resistant.

A: Specimen Type

B: Treatment of the patient when clinical sample was isolated (only for *P. aeruginosa*)

C: Broth microdilution method.

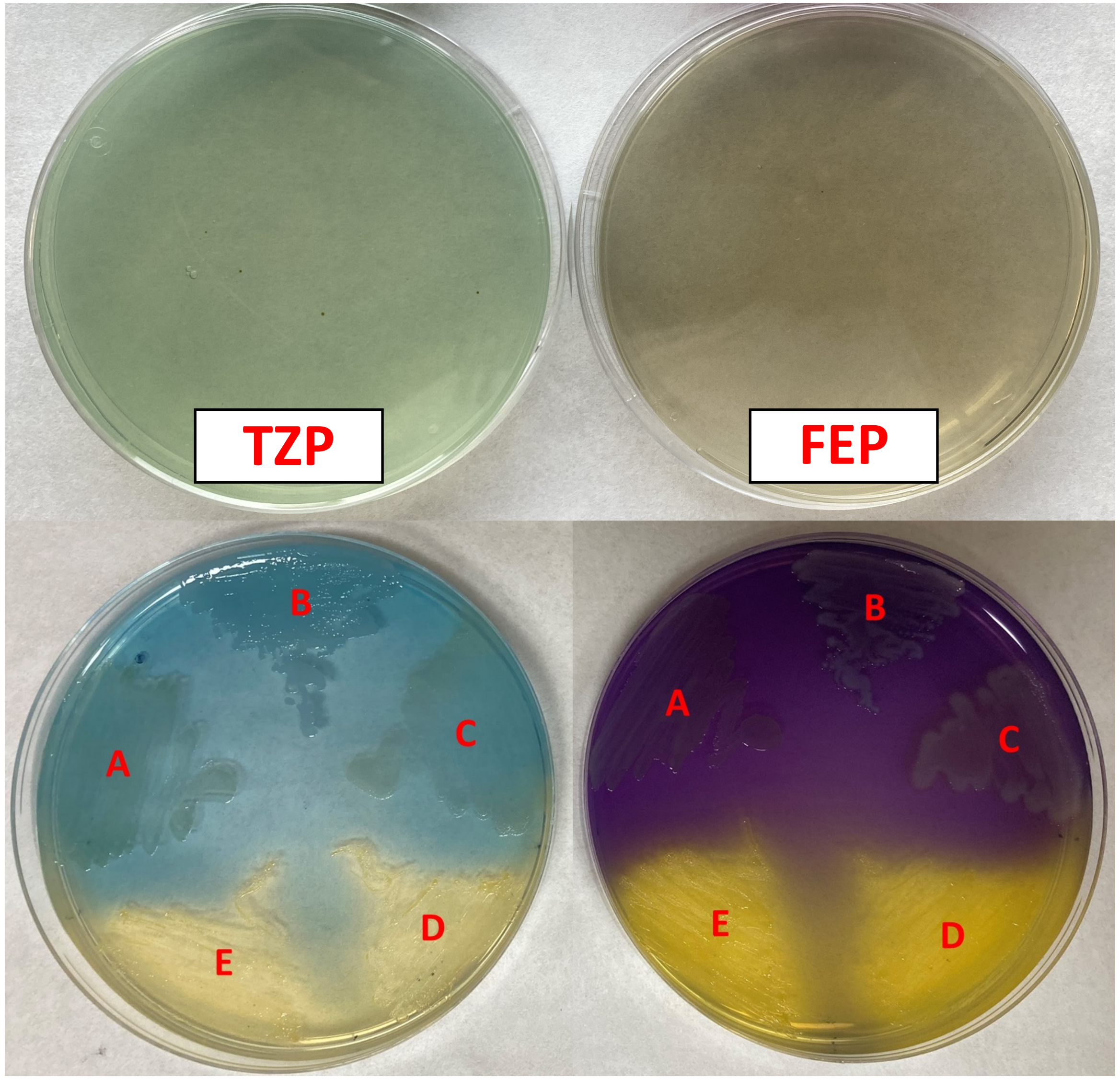

Supplementary Figure 1. Colour change of the media with different fermenter and non-fermenter species. Upper plates pictures correspond to the original colour of each section of the bi-plate before bacterial growth. (A) *Stenotrophomonas maltophilia*, (B) *Acinetobacter baumannii*, (C) *Pseudomonas aeruginosa*, (D) *Klebsiella pneumoniae*, and (E) *Escherichia coli*.
